# Supplementary material for: The Global COVID-19 Pandemic Experience: Innovation Through Environmental Assessment and Seropositivity Surveillance
Source: Int J Environ Res Public Health. 2025 Jul 18;22(7):1145. doi: 10.3390/ijerph22071145 (PMC12294177; doi:10.3390/ijerph22071145)
Supplement: Supplementary file 1 [file ijerph-22-01145-s001.zip › ijerph-3645409-supplementary.pdf]

Supplementary material

Supplemental text, tables and figures

1. Extrapolation of cumulative mortality to final pandemic status
2. Trends in infection vs. fatality
3. Determination of final (01-01-2023) COVID-19 annual mortality rate by graphical method
4. Cumulative COVID-19 deaths as of Aug 30, 2023

1 <u>Extrapolation of cumulative mortality to final pandemic status</u>

Extrapolation to ultimate pandemic mortality burden (references pertain to published paper)

The different time courses of cumulative COVID-19 mortality among countries together with vaccination status permits estimation of the likely final COVID-mortality burden in countries by 2025. The proportion vaccinated[11,12] and an estimate of seropositivity at the end of 2022 together with assumptions regarding mortality protection factors afforded by vaccination or natural immunity permits a calculation of the population remaining at risk for COVID-19 mortality at that time. Of interest for this analysis is the reciprocal of the IFR, which would be the ratio of new infections to new fatalities in the same period (the *fatality infection ratio,* FIR=1/IFR). Applying the final COVID-19 mortality rate in 2022 (obtained, graphically, as the slope of the cumulative mortality rate per million; Figs. S1, S2)[12] to a population for two more years making the conservative assumption that the levels of immunity (natural and vaccinated) don't change (Scenario 1), would produce an upper bound on the additional COVID-19 deaths after 2022. Assuming the pandemic will have resolved to a steady-state endemic condition by the end of 2024 (Scenario 2) with a) mortality rates of 10% of the initial 2023 rates (i.e., final 2022 rates), and b) the average COVID-19 mortality rate over the two-year period being the mean of the initial rate and final rate during 2023, 2024, would produce a lower over-estimate of the final mortality burden. Finally using a) the FIR from the observed mortality to estimate natural seropositivity from the observed mortality and b) the vaccination proportion, as of the end of 2022, and assuming that seropositivity and vaccination approach 95% by 2025 (Scenario 3) permits a third estimate of ultimate pandemic mortality burden. In Scenario 3, extreme FIR values in countries where the available FIR data was based on early seropositivity surveys (Italy, Taiwan and Japan) were replaced as follows: a) Italy (FIR=444) assigned UK's FIR: 94; b) Japan (FIR=42857) and Taiwan (FIR=1724) were replaced by value from S Korea: 266.

For Scenario 3 the population-at-risk proportion (PaR) at the end of 2022 taking into account the population unvaccinated or with zero seropositivity was calculated as follows:

$$PaR(01\text{-}01\text{-}2023) = 1-(SP \times (1-\%VAC/100) \times 0.90 + (1-SP) \times \%VAC/100 \times 0.95 + SP \times \%VAC/100 \times 0.98)$$

where SP = natural COVID-19 seropositivity; SP = cumulative mortality per million × FIR /1,000,000
%VAC = percent completing full original vaccination protocol
fatality protection factor (PF) for prior infection was 90%
fatality protection factor for vaccination was 95%

fatality protection factor for both vaccination and prior infection was 98%

This expression assumes that the FIR measured previously (in most cases prior to vaccination) applies in the future. At the end of 2024 the PaR was specified to be:

$PaR(01\text{-}01\text{-}2025) = 1-(0.95\times0.05\times0.90 + 0.05\times0.95\times0.95 + 0.95\times0.95\times0.98)$

where final SP was assumed = 95% and %VAC = 95%.

The mortality rate in an unprotected population (TMR) was calculated from the observed final COVID-19 mortality rate (OMR, derived graphically) at 01-01-2023 as follows:

$TMR = OMR/PaR(01\text{-}01\text{-}2023)$

In predicting mortality during 2023-2024 (Scenario 3) the rate TMR was applied to the mean of PaR(01-01-2023) and PaR(01-01-2025):

$$\text{deaths (2023-2024)} = \text{population} \times 2\text{ yr} \times TMR \times (PaR(01\text{-}01\text{-}2023) + PaR(01\text{-}01\text{-}2025))/2\ .$$

In the case of Japan, where abrupt increases in the COVID-19 mortality rate were observed in Jan. and July of 2022 (due to COVID-19 variants and/or altered COVID-19 prevention practices), estimates of the additional deaths expected were also calculated based on the cumulative deaths per million and the COVID-19 mortality and vaccination rates observed prior to Jan. or July of 2022. This prediction assumes that those COVID-19 mortality rates would be maintained with adjustments in public health practices dealing with, for example, new COVID-19 variants.

Alternate scenarios:

Scenario 1: apply final mortality rate to total population assuming constant population at risk (fixed seropositivity).

Scenario 2: apply average mortality rate assuming mortality rate @ 1-1-2023 rate declines to 10% by 1-1-2025.

Scenario 3: apply final mortality rate in unprotected (@ 1-1-2023) assuming subpopulation at high risk (unvaccinated or zero seropositivity) with *PF*(*seropos*.) = 0.80, *PF*(*vacc*) = 0.90 and *PF* = 0.98 for both natural and vaccine immune protection.

Scenario 4: apply final true mort rate (@ 1-1-2023) assuming subpopulation at high risk (unvaccinated and zero seropositivity) declines to almost zero by 1-1-2025 (Scenario 3) but with FIR set = 100 for poor performing countries and = 200 for well-performing countries.

Scenario 5: apply final true mort rate (@ 1-1-2023) assuming subpopulation at high risk (unvaccinated and zero seropositivity) declines to almost zero by 1-1-2025 (Scenario 3) but with FIR set = 100 for poor performing countries and = 200 for well-performing countries, with *PF*(*seropos*.) = 0.80, *PF*(*vacc*) = 0.90 .

Scenario 6: apply final true mort rate (@ 1-1-2023) assuming subpopulation at high risk (unvaccinated and zero seropositivity) declines to almost zero by 1-1-2025 (Scenario 3) but with FIR set = 100 for poor performing countries and = 200 for well-performing countries, with *PF*(*seropos*.) = 0.80, *PF*(*vacc*) = 0.90 (Scenario 5) and with PF = 0.95 for both natural and vaccine immune protection (change from 0.98).

Results

With prediction of future COVID-19 deaths until 2025 under Scenario 1, the four Western countries (U.S, UK, Italy and Germany) had an estimated final mortality burden ranging 2977 to 4275 per million, and the East Asian/New Zealand countries ranged 983 to 2437 per million (Table S4). Under Scenario 2 the burdens were smaller and in the case of Japan, when derived from two earlier starting dates and rates, were considerably lower: 345 and 273 per million (Table S5). Accounting for natural seropositivity (limited representative survey data) and vaccination and replacing extreme FIR values in countries where the available FIR data was based on early seropositivity surveys (Scenario 3, Table S6) the PaR varied from 0.126 to 0.239 indicating that by the end of 2022 in the countries analyzed more than 75% of people had immunologic protection. Under Scenario 3 for Japan but calculating from two earlier starting dates prior to relaxation, the ultimate burdens were still lower: 348 and 276 per million. Thus, the mortality experience for Japan predicted from the earlier dates was exceptionally superior to that of the major countries as well as for the other East Asian countries and New Zealand analyzed here (Table S6). This same calculation was repeated replacing the country-specific FIR estimates, which could represent non-representative surveys with respect to population sample and time period, with a uniform FIR: set = 100 for poorly performing countries and = 200 for well-performing countries. The results were very similar (Scenario 4). Assuming lower mortality protection factors for seropositivity (0.8) and vaccination (0.9) (Scenario 5), and reducing the PF for joint natural and vaccine protection to 0.95, produced only slightly higher final mortality burdens (Scenario 6). The predicted cumulative COVID-19 mortality results from the six scenarios are displayed in Table S7.

Table S1 COVID-19 mortality rates at end of 2022 and predicted final pandemic mortality burden by start of 2025: Scenario 1

Country[11]	Pop (M)	Cum. mort. /M @ end 2022	Observed annual mort. rate /M @ end 2022[1]	% Vacc. @ end 2022	Additional deaths/M @ end 2024	Predicted pandemic cum. mort. @ start of 2025 /M	Predicted pandemic deaths @ start of 2025
US	329	3230	367	68.6	733	3963	1303937
UK	67.1	3159	367	76.5[1]	733	3892	261176
Italy	59.5	3128	573	81.3	1147	4275	254343
Germany	83.2	1937	520	76.5	1040	2977	247686
Taiwan	23.6	639	460	86.3	920	1559	36792
S Korea	51.7	623	393	86.3	787	1410	72880
Japan	126.3	464	986	83.2	1973	2437	307835
New Zealand	5.1	450	267	79.8	533	983	5015

Scenario 1: apply final mortality rate to total population assuming constant population at risk (fixed seropositivity).

1 Based on 9-month interval (Mar 31, 2022 to Dec 31 2022) to facilitate retrieval of rate from graphical presentation (manuscript Figs. 3, 4)

Table S2 COVID-19 mortality rates at end of 2022 and predicted final pandemic mortality burden by start of 2025: Scenario 2

Country[11]	Pop (M)	Cum. mort rate/M @ end 2022	Observed annual mort. rate /M @ end 2022[1]	% Vacc. @ end 2022	Additional deaths/M @ end 2024	Predicted pandemic cum. mort. @ start of 2025 /M	Predicted pandemic deaths @ start of 2025
US	329	3230	367	68.6	404	3634	1195533
UK	67.1	3159	367	76.5[1]	404	3563	239066
Italy	59.5	3128	573	81.3	631	3759	223688
Germany	83.2	1937	520	76.5	573	2510	208808
Taiwan	23.6	639	460	86.3	507	1146	27037
S Korea	51.7	623	393	86.3	433	1056	54606
New Zealand	5.1	450	267	79.8	294	744	3793
Japan	126.3	464	986	83.2	1087	1551	195852
Japan[2]	126.3	235	80	82.0	110	345	43560
Japan[3]	126.3	145	80[4]	81.0	128	273	34527

Scenario 2: apply average mortality rate assuming mortality rate @ 1-1-2023 rate declines to 10% by 1-1-2025.

1 Based on 9-month interval (Mar 31, 2022 to Dec 31 2022) to facilitate abstraction of rate from graphical presentation (manuscript Figs. 3, 4)

2 Prediction based on cumulative deaths and mortality rate as of July 24 2022

3 Prediction based on cumulative deaths and mortality rate as of Jan 27 2022

4 Rate prior to Feb 2022 was virtually zero; used rate prior to July 24 2022

Table S3 Predicted pandemic mortality burden by 2025: Scenario 3

Country	Pop (M)	Cum. mort. rate/M @ end 2022	Observed annual mort. rate /M @ end 2022[1]	% Vacc. @ end 2022	durF	Avg. FIR	Proport. high risk @ end 2022	Annual mort. rate /M unprotect. @ end 2022	Additional deaths/M 2023-24	Predicted pandemic cum. mort. @ start of 2025 /M	Predicted pandemic deaths @ start of 2025
US	329	3230	367	68.6	2.67	227[2]	0.126	2909	448	3678	1,209,982
UK	67.1	3159	367	76.5[3]	2.67	94	0.204	1801	417	3576	239,952
Italy	59.5	3128	573	81.3	2.67	94[4]	0.171	3352	667	3795	225,800
Germany	83.2	1937	520	76.5	2.67	75	0.239	2173	581	2518	209,489
Taiwan	23.6	639	460	86.3	2.67	266[5]	0.155	2971	543	1182	27,893
S Korea	51.7	623	393	86.3	2.67	266	0.155	2530	464	1087	56,195
S Korea[6]	51.7	623	393	86.3	2.67	266	0.155	2530	-	720	37,224
New Zealand	5.1	450	267	79.8	2.67	200	0.223	1193	300	750	3,825
Japan	126.3	464	986	83.2	2.67	266[5]	0.188	5251	1133	1597	201,756
Japan[6]	126.3	235	80	82.0	-	266	0.209	382	-	650	82,095
Japan[7]	126.3	235	80	82.0	3.33	266	0.209	382	113	348	43,966
Japan[8]	126.3	145	80	81.0	3.89	266	0.223	359	131	276	34,882

Scenario 3: apply final mortality rate in unprotected (@ 1-1-2023) assuming subpopulation at high risk (unvaccinated or zero seropositivity) declines to almost zero by 1-1-2025, with *PF*(*seropos.*) = 0.80, *PF*(*vacc*) = 0.90 and *PF* = 0.98 for both natural and vaccine immune protection.

1 Annual rate based on 9-month period (Mar 31, 2022 to Dec 31 2022) to facilitate abstraction of rate from graphical presentation (manuscript Figs. 3, 4)

2 U.S. FIR based on *IFR*=0.00441 derived from Clarke et al.[8]

3 vaccination status for England

4 FIR for Italy (444, 05/10/2020) replaced by value from UK: 94

5 FIR for Japan (42,857, very early in pandemic, 04/07/20) and for Taiwan (1724, 07/15/2020) replaced by value from S Korea: 266 (11/01/2020)

6 Prediction based on cumulative deaths as of 01-01-23 and final mortality estimated from trend over 2022-2023 for Japan and S. Korea

7 Prediction based on cumulative deaths and mortality rate as of July 24, 2022

8 Prediction based on cumulative deaths and mortality rate as of Jan 27, 2022

Table S4 Final COVID-19 pandemic mortality burden by start of 2025, predicted under alternate scenarios based on annual mortality rates observed at end of 2022, and observed

Country	Final cumulative pandemic mortality per million (2020 – 2024) predicted in Scenarios 1-6 and observed at start of 2025						
	1	2	3	4	5	6	Observed, start of 2025
	Western countries						
US	3963	3634	3678	3648	3642	3675	3548
UK	3892	3563	3576	3590	3581	3621	3404
Italy	4275	3759	3795	3821	3801	3872	3345
Germany	2977	2510	2518	2537	2526	2578	2081
	East Asian and New Zealand countries						
Taiwan	1559	1146	1182	1199	1180	1239	-
S Korea	1410	1056	1087	1102	1085	1136	694
New Zealand	983	744	750	759	753	780	598
Japan	2437	1551	1597	1630	1600	1712	876
Japan[1]	-	345	348	351	349	359	876
Japan[2]	-	273	276	280	277	289	876

1 Prediction based on cumulative deaths and mortality rate as of July 24, 2022

2 Prediction based on cumulative deaths and mortality rate as of Jan 27, 2022

Scenario 1: apply final mortality rate to total population assuming constant population at risk (fixed seropositivity).

Scenario 2: apply average mortality rate assuming mortality rate @ 1-1-2023 rate declines to 10% by 1-1-2025.

Scenario 3: apply final mortality rate in unprotected (@ 1-1-2023) assuming subpopulation at high risk (unvaccinated or zero seropositivity) declines to almost zero by 1-1-2025, with *PF*(*seropos*.) = 0.80, *PF*(*vacc*) = 0.90 and *PF* = 0.98 for both natural and vaccine immune protection.

Scenario 4: apply final true mort rate in unprotected (@ 1-1-2023) assuming subpopulation at high risk (unvaccinated and zero seropositivity) declines to almost zero by 1-1-2025 (Scenario 3) but with FIR set = 100 for poor performing countries and = 200 for well-performing countries.

Scenario 5: apply final true mort rate in unprotected (@ 1-1-2023) assuming subpopulation at high risk (unvaccinated and zero seropositivity) declines to almost zero by 1-1-2025 (Scenario 3) but with FIR set = 100 for poor performing countries and = 200 for well-performing countries, with *PF*(*seropos*.) = 0.80, *PF*(*vacc*) = 0.90 .

Scenario 6: apply final true mort rate in unprotected (@ 1-1-2023) assuming subpopulation at high risk (unvaccinated and zero seropositivity) declines to almost zero by 1-1-2025 (Scenario 3) but with FIR set = 100 for poor performing countries and = 200 for well-performing countries, with *PF*(*seropos*.) = 0.80, *PF*(*vacc*) = 0.90 (Scenario 5) and with *PF* = 0.95 for both natural and vaccine immune protection (change from 0.98).

2 Trends in infection vs. fatality

If the ratio of the infection incidence rate to the COVID-19 death rate were independent of the virus dose from the exposure episode causing the infection then the FIR for countries or regions (Tables S5, S6) should not depend on the average levels of virus exposure for those areas. In the countries for which there are overall seropositivity survey estimates and concurrent cumulative fatality data, the FIR appears

to decrease by an order of magnitude with increasing observed average COVID-19 mortality rate (the surrogate for COVID-19 relative exposure) (Fig. S1) but this is weak support for the hypothesis that FIR is higher at lower COVID-19 relative exposure due to the limitations in both the FIR and virus exposure data. The same trend is observed for regions within countries (Fig. S2). Stronger support comes from observing that during 2020 the rather widely variable FIR values at the average exposure surrogate for high mortality countries (U.S, UK, Italy, Spain, Sweden: FIR=179 (geometric mean); relative exposure=119) were considerably less than for the low mortality countries (Taiwan, New Zealand, S Korea, Japan: FIR=1466 (geometric mean); relative exposure=0.47) (Table S5). Replacing the very high FIR value for Japan – 42,800 – from early in pandemic (04-07-20) with that of S Korea (268) still yields a much higher FIR=397 (Table S5).

Table S5 Seropositivity estimates for selected countries

Country	DatesP	sP	Cmort/M	IFR	FIR	Tp	avgX
High average-exposure countries							
UK	01/15/2021	0.153	1647.8	0.01077	93	11.5	143
US	12/31/2020	0.31	1036.3	0.00334	299	11.0	94
US	02/01/2022	0.60	2648.5	0.00441	227	24.0	110
Italy	05/10/2020	0.231	514.8	0.00223	448	3.3	156
Spain	07-06-2020	0.052	597.1	0.01148	87	5.2	114
Sweden	07-23-2020	0.073	537.2	0.00736	136	5.7	94
Geometric mean FIR: 179 Mean avgX: 119							
Low average-exposure countries							
Japan/Kobe	4/7/2020	0.033	0.77	0.00002	42800	2.2	0.35
New Zealand	12/16/2020	0.001	5.01	0.00501	200	10.5	0.48
South Korea	11/1/2020	0.0024	8.96	0.00373	268	9.0	1.00
Taiwan/hosp	7/15/2020	0.0005	0.29	0.00058	1695	5.5	0.053
Geometric Mean FIR: 1466 Mean avgX: 0.47							
Low average-exposure countries (substituting S Korean FIR for Japan)[1]							
Geometric mean FIR: 397 Mean avgX: 0.47							

DatesP – date of survey sP – seropositivity Cmort/M – cumulative mortality per million
IFR – infection fatality ratio FIR – fatality infection ratio (=1/IFR) Tp – time from April 01, 2020 until survey (months) avgX – average relative virus exposure based on attained pandemic duration and cumulative mortality rate: avgX = (Cmort/M) / Tp

1 replacing very high FIR value for Japan – 42800 – from early pandemic (04-07-20) with FIR of South Korea (268)

Table S6 Sub-country regions: seropositivity estimates in 2020 prior to vaccination in order of survey date

Country	Date sP	sP	Pop	Cum mort /M	SDR	IFR	FIR
US/California (Santa Clara)	03-20-2020	0.028	1.92M	46.9	43.7	0.0017	588
US/NYC (Queens)	06-15-2020	0.68	2.27M	1419	36.7	0.0021	476
Germany/Reutlingen,1	07-15-2020	0.026	237154	337.3	4.1	0.013	77
Germany/Freiburg,1	08-15-2020	0.015	412129	366.4	3.0	0.024	42
Germany/Aachen	09-15-2020	0.023	470785	218.8	4.9	0.010	100
Colombia/Monteria	10-14-2020	0.55	500000	3170	11.0	0.0058	172
Germany/Reutlingen,2	10-15-2020	0.028	237154	341.6	3.5	0.012	83
Germany/Osnabrück	10-15-2020	0.013	434567	211.7	2.6	0.016	63
Germany/Freiburg,2	11-15-2020	0.024	412129	414.9	2.2	0.017	59
Germany/Magdeburg	11-15-2020	0.024	201596	74.4	5.1	0.003	333
Germany/Tirschenreuth	11-22-2020	0.092	64643	2289	3.67	0.023	43
SA/Gauteng	12-15-2020	0.684	15.5M	415.7	45.6	0.00061	1639
India/Ahmedabad	12-31-2020	0.176	8.1M	146.2	87.1	0.00083	1205

Date sP – date of survey sP – seropositivity Pop – population Cum mort /M – cumulative COVID-19 mortality as of survey date SDR – (case fatality rate, CFR)/IFR IFR – infection fatality ratio FIR – fatality infection ratio

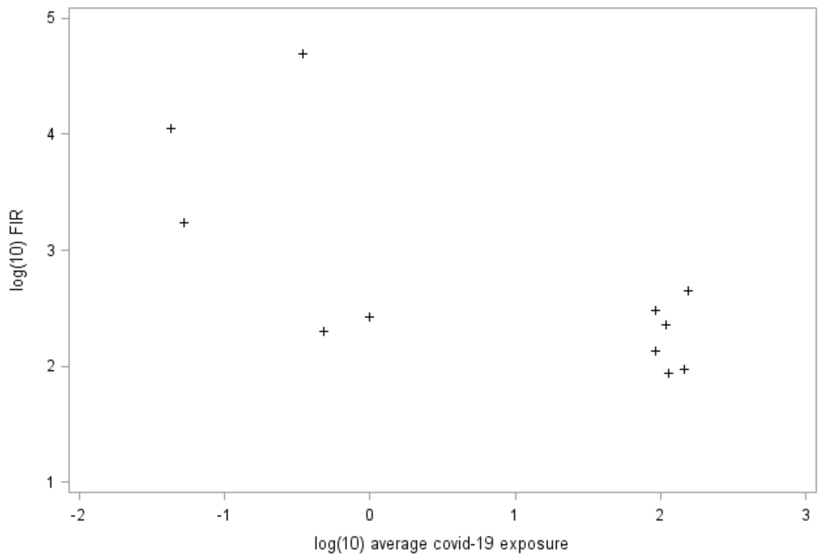

Figure S1 Fatality infection ratio (FIR) trend on average relative COVID-19 exposure intensity by country (based on mortality)

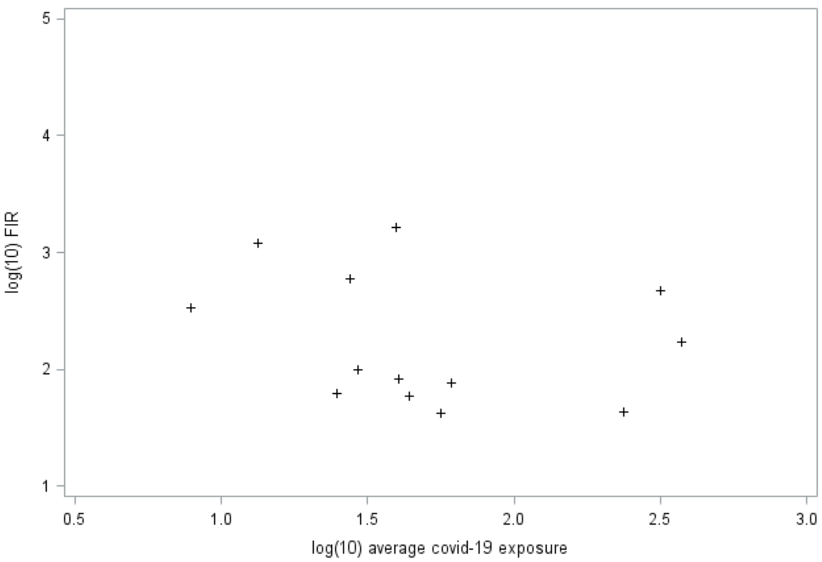

Figure S2 Fatality infection ratio (FIR) trend on average relative COVID-19 exposure intensity by region (based on mortality)

3 Determination of final (01-01-2023) COVID-19 annual mortality rate by by graphical method

Figure S3 Slope abstraction: Taiwan example

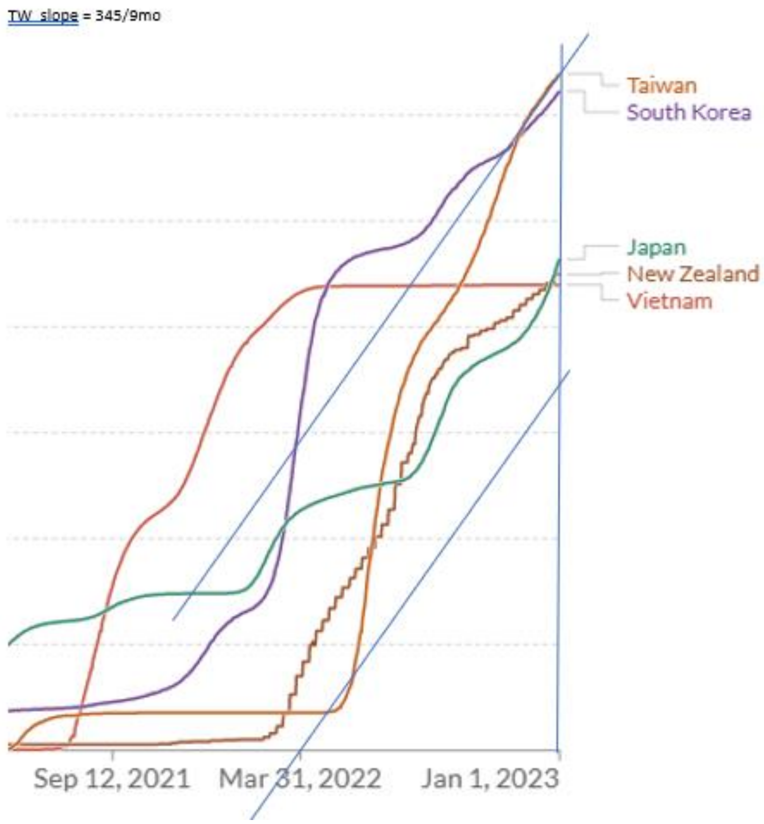

Graph strata: 100/M progression

Figure S4 Slope abstraction: US example

**

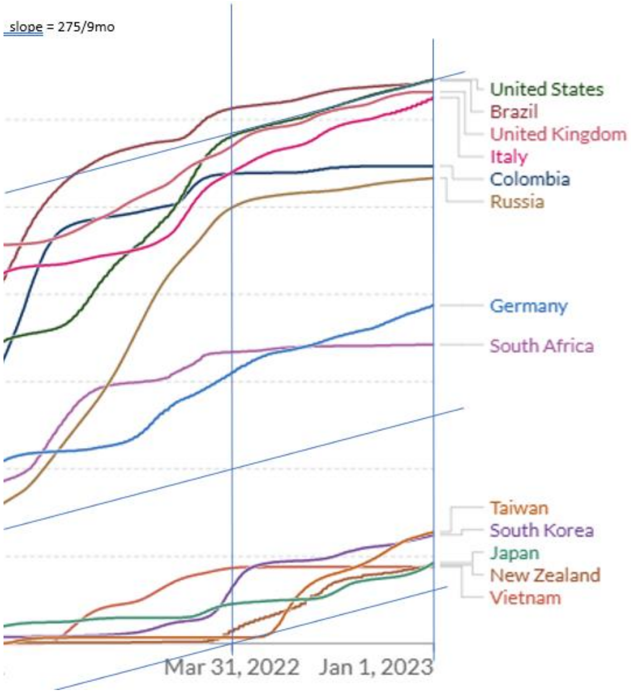

Graph strata: 500/M progression

4 Cumulative COVID-19 mortality rate as of Aug 30, 2023

Figure S5 Cumulative COVID-19 deaths per million for selected countries as of Aug 30, 2023 (data from Johns Hopkins University CSSE[1], displayed by software at OurWorldInData.org[2] and provided under Creative Commons BY open access license, and now under WHO auspices; data from Taiwan no longer compiled)

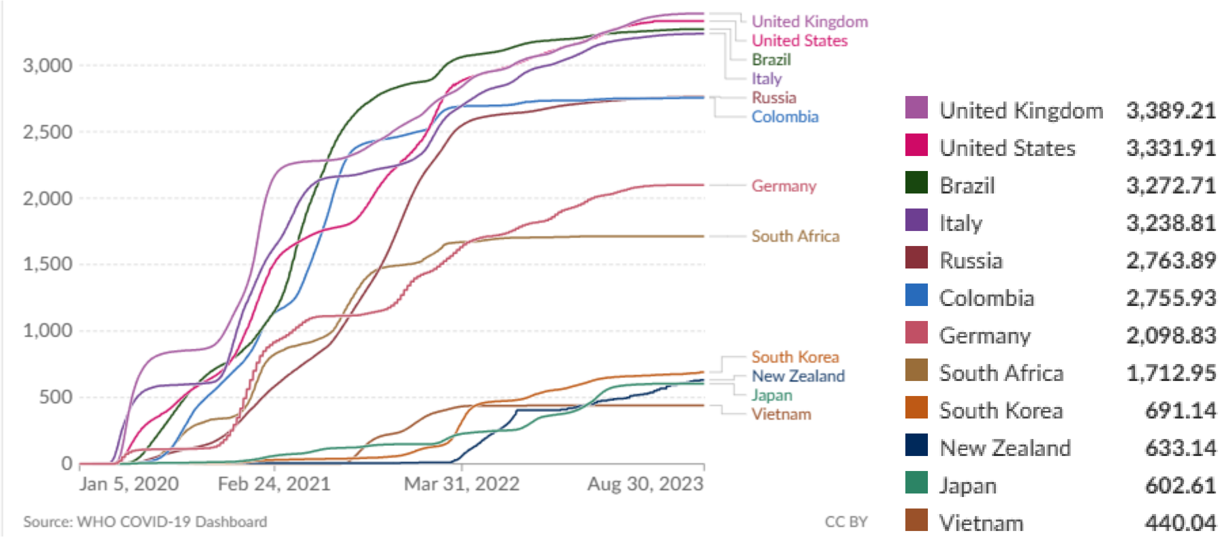

References

1. Coronavirus Resource Center, Center for Systems Science and Engineering (CSSE), Johns Hopkins University, Baltimore MD; https://coronavirus.jhu.edu/about , https://systems.jhu.edu/research/public-health/ncov/
2. Mathieu E, Ritchie H, Rodés-Guirao L et al. (2020). Coronavirus Pandemic (COVID-19). Global Change Data Lab: Published online at OurWorldInData.org: https://ourworldindata.org/coronavirus
